# “Put your personality into the call”: A qualitative interview study illuminating strategies for improving men’s engagement on crisis helplines

**DOI:** 10.1186/s12889-024-19242-x

**Published:** 2024-06-27

**Authors:** Katherine Trail, Kieran O’Gorman, Zac Seidler, John Oliffe, Tara Hunt, Simon Rice

**Affiliations:** 1 Orygen, Parkville, Melbourne, Australia; 2 Centre for Youth Mental Health, The University of Melbourne, Melbourne, VIC Australia; 3 School of Nursing, University of British Columbia, Vancouver, Canada; 4 Department of Nursing, The University of Melbourne, Melbourne, VIC Australia; 5 Lifeline Research Foundation, Lifeline Australia, Sydney, NSW Australia

**Keywords:** Telephone crisis helpline, Suicide prevention, Men’s health, Masculinities, Service engagement, Crisis intervention

## Abstract

**Background:**

Crisis telephone helplines are an integral part of community suicide prevention. Despite high male suicide rates, men’s experiences of these services are poorly understood. The current study explored men’s perspectives of their interactions with helpline counsellors to understand how their engagement on helplines can be enhanced.

**Method:**

Sixteen men (19–71 years) who had previously used a mental health or crisis helpline in Australia completed individual semi-structured interviews about their experiences. Data were analysed using interpretive descriptive methodologies.

**Results:**

Two themes derived from the data related to how men engaged with counsellors on helpline services. First, men emphasized the importance of helpline counsellors creating and maintaining an authentic connection across the call, providing suggestions for strategies to secure connection. Second, men discussed how counsellors can facilitate outcomes through offering space for their narratives and aiding in referrals to other support services when required.

**Conclusions:**

Findings highlight the value of crisis helplines for men’s suicide prevention services while identifying target areas to improve engagement. We discuss implications for the findings including suggestions for gender-sensitive care within crisis helplines.

**Supplementary Information:**

The online version contains supplementary material available at 10.1186/s12889-024-19242-x.

## Introduction

Crisis helplines are an essential component of community suicide prevention, providing accessible, anonymous, and cost-effective support to individuals experiencing distress and/or crisis [1]. Men’s experiences of crisis helplines are under-researched, despite the fact that men are three times more likely to die by suicide than women in Australia and other high-income countries [2, 3]. This long-standing statistic is often attributed in part to men’s reluctance for help-seeking, with decades of research belabouring the ways in which traditional masculinity precludes men from seeking or accessing support for fear of being perceived as fragile or weak, and therefore feminine [4–7]. As a result, shame, embarrassment and stigma often accompany and further fuel men’s experiences of mental distress [8–10].

However, the historical focus on gender and sex differences in help-seeking has in recent years been criticised for failing to comprehensively consider the diversity of men’s help-seeking attitudes and experiences [7]. While norming men’s help-seeking is important, the experiences of men who *do* seek support is especially salient in affirming men’s help-seeking practices [11]. Instead, investigating *how* men experience help-seeking within specific contexts is central. Recent focus has shifted the onus to health services to ensure they are effective and engaging to men, with suggestion this might best be done by incorporating a masculinities model into care [12]. Put simply, this would involve increasing understanding of how men’s alignments to masculine ideals interact with experiences of distress and help-seeking, allowing health care counsellors to recognise and work with a plurality of masculinities to strengthen engagement [12–14]. Proponents of this model argue that that a masculinities model of mental health care is needed to not only encourage men to seek help, but also to *retain* them in care by prioritising their needs and preferences, consistent with the principles of person-centred care [12]. As prominent entry-points and sources of referral to mental health care, this is a salient concern for crisis helplines.

Men represent at least 40% of callers to Australia’s largest crisis helplines, with this proportion increasing in recent years indicating the importance of ensuring these services are able to effectively engage with men and respond to their distress [15]. This necessity is heightened by the fact that helplines reduce many structural barriers to accessing care [1] and thus may be servicing a population of men that are not willing or able to access other forms of mental health care. Helplines are normally staffed by paid and volunteer professionals and paraprofessionals who are trained in crisis support [16, 17], referred to in this paper as counsellors.

The helpline context presents several unique challenges for counsellors in responding to and supporting men in distress. Helpline counsellors must establish rapport, assess suicide risk, and provide support in a brief time period and without access to information (e.g., demographic characteristics and non-verbal cues such as body language) that is available in other clinical contexts. Although anonymity, accessibility and caller control over the interaction may reduce some discomfort related to the experience of emotional vulnerability, some research suggests that masculine socialization continues to be a barrier to engagement in this context [18]. Studies into men’s behaviours using physical health helplines indicate that men frequently call at the behest of a (usually female) loved one, and often minimize or downplay the severity of their own ailments [19, 20]. The extent to which this applies to crisis helplines is unclear.

### Men and crisis helplines

Previous research examining men’s experiences of helplines is limited. While systematic reviews highlight the effectiveness of helplines in reducing distress and proximal suicide risk, little is known about their impact on men specifically due to methodological constraints in collecting caller information such as gender [21, 22]. Where gender demographics are collected, men are underrepresented in the research; with under 30% of participants across 25 studies reported as male [23]. One study of a Canadian suicide prevention helpline found that men’s suicidality improved less frequently than women across a call, and that men on average had poorer outcomes post-call than women [24]. Service expectations also seemingly differ by gender, with men being less likely to endorse ‘expecting to feel less alone’ and ‘feeling more connected’ during a call to Lifeline than women [25].

Some research indicates that the ways in which helpline staff engage with and respond to male callers are influenced by expectations about the way men experience distress, and the type of care they require. For example, one study found that helpline counsellors frequently attempted to provide practical advice and solutions despite male callers indicating a desire to simply talk about their concerns, indicating a potential unmet need for supportive listening [26]. Further, helpline counsellors may be more inclined to implement emergency procedures with male callers due to perceptions of increased suicide risk in men [27], and associate risk-taking or recklessness with suicidality in men only [28]. The above research suggests that helpline services may function uniquely for male callers, though little is known about how men perceive and experience these services. Drawing attention to this under-researched area, our team recently conducted a survey including qualitative responses from 92 Australian men regarding their crisis helpline experiences [18]. Men’s responses gave insight into a diversity of needs including de-escalation, emotional support and the provision of resources or referrals, and identified aspects of helpline interactions that impacted their level of engagement on the call; however in-depth exploration is required to further elucidate experiences.

### The current study

Despite the critical importance of crisis helplines as a support service for men experiencing distress and suicidality, there is a paucity of knowledge regarding men’s subjective experiences of helplines. To address this gap, the current study uses in-depth interviews to gain understandings about men’s perspectives on and experiences with crisis helplines. Specifically, the current study places emphasis on *how* men experience the interactions with crisis helpline counsellors, and the elements of the interaction that impact their level of engagement with those service.

## Methods

The study was approved by (blinded for review) Human Research Ethics Committee (ethics ID: 2022-22987-30261-7). The study is reported according to the SRQR guidelines for reporting qualitative research (see additional file 1) [29].

### Participants

Men aged ≥ 18 who had previously used a crisis helpline in Australia were eligible to participate in the study. Eligible services included any service that offered one-off and anonymous phone support related to mental health or wellbeing concerns (i.e., not a medical helpline, an information only line, or a service that offered continuing care).

### Procedure

The study took place during the first half of 2022. Participants were recruited via advertisements posted on university staff and student newsletters, social media (Twitter, Facebook, LinkedIn), and through investigator networks. Advertisements included brief information about the study aim, the inclusion criteria, and option to contact the first author or register interest via an online form securely hosted on Qualtrics. Interested participants were contacted via email by the first author who arranged a time to meet via Zoom or phone to further explain the study, assess eligibility and obtain informed consent.

Interviews were conducted via Zoom between February and April 2022. The first author conducted interviews from her place of work in Melbourne, Australia, while participants joined from a place of their choosing. The interviewer confirmed that participants could comfortably access the software prior to commencing the interview.

A semi-structured interview guide was used which include open ended questions related to men’s experiences of and attitudes towards helpline services. Specifically, questions focused on the period leading up to a decision to contact a crisis helpline and the experience of the interaction with the counsellor on the call. For this study, key interview questions included, *Please tell me about your experience of calling a helpline? What worked well for you when using a helpline? What could have been improved about your experience using a helpline?* (See additional file 2 for full list of questions related to men’s interaction with their helpline counsellor). Participant demographics and helpline use information was collected via an online form hosted by Qualtrics following informed consent procedures.

All participants were offered an opportunity to review their interview transcript and provide additional information or redact specific details. Only one participant took up this offer, who returned the transcript with no changes. Participants were reimbursed with an e-gift card of $36 AUD. Audio recordings were transcribed and accuracy checked by KT. Transcripts were then deidentified prior to analysis and participants were allocated a pseudonym by the researchers. Transcripts and recordings were kept on password-protected, secure servers only accessible by the researchers.

Given the sensitive nature of the topics discussed in interviews, a clinical safety protocol was developed in anticipation that some participants might become distressed or express suicidal ideation (see additional file 4).

### Researcher characteristics

All interviews were conducted using Zoom by the first author (KT), a cisgendered white woman in her mid-20s. She had an honours degree in psychology and had previously conducted qualitative interviews with men regarding mental health. She did not have personal experience of crisis helpline use and was able to bracket in taking a discovery approach to learning about men’s experiences with the services. She completed memos during and after each interview to reflect on her assumptions or preliminary thoughts about the interviews. KT was also the primary data analyst. Analysis was discussed with the wider research team consisting of four male researchers with expertise in qualitative research methodologies, masculinities and men’s health research, and a female researcher in the helpline sector, allowing for a diversity of perspectives to be included in the final analysis presented.

### Theoretical underpinnings

This study was developed with the purpose of informing a training program for helpline counsellors at Lifeline Australia to enhance counsellor skills in engaging with male callers. Data were analysed using interpretive description analysis [30] according to the approach described by Thomspon Burdine and colleagues [31]. Interpretive description is an inductive analysis approach that allows for practical implications to be drawn from patterns identified in the data. In this case, data were analysed with the specific focus to identify topics that could relate to counsellor skills in engaging and communicating with male callers.

The researchers took a constructivist approach to data collection and analysis, whereby subjective reality is cocreated through the interaction between researcher and participant. This approach was adopted because the research was more concerned with participants’ lived experiences than uncovering one objective reality or truth. The researchers drew upon their knowledge of masculinities frameworks [13] and work on men’s engagement with mental health services [32] when conducting interviews and analysis.

### Data analysis

Data were uploaded to NVIVO-12 (QSR, 2018) and data collection and analysis occurred concurrently. First, data familiarisation was achieved by repeated reading of full interview transcripts. Basic concepts were then identified through open coding of the transcripts. A template analysis was also used, in which a subsection of the data was coded, and a preliminary thematic structure developed. This structure was applied iteratively to the remaining data set, with revisions as necessary. The first 10 interviews were included in the initial template, which was developed in consultation with the wider research team in meetings (see additional file 3). The remaining six interviews were then coded onto this template, with potential changes or differences noted. Refinements were discussed with the research team prior to a final thematic structure being developed. This allowed for a record of changes made during the analysis process. Finally, the subthemes were subsumed to reflect two broad themes in the data, *(In)authenticity: securing connection on the call* and *Men’s expectations of the counsellor’s role in facilitating outcomes*, both carrying practical implications for helpline service delivery. The thematic structure was further refined during the writing and editing of this manuscript, and theme names and accompanying quotes decided on by the research team. Points of disagreement were discussed by the research team and resolved by consensus. Recruitment was ceased based on the researcher’s judgement of the depth of the data collected and the weight of the developed thematic structure.

## Results

Twenty-seven men completed the online form. Of these, five did not meet the inclusion criteria, five could not be contacted, and one declined to participate. Sixteen men aged between 19 and 71 years (M = 37.94, SD = 15.18) completed interviews (see Table 1). The majority of participants (68.7%) had used a crisis helpline in the last year, and most (56.2%) had used more than one service with Beyond Blue and Lifeline equally the most used (50%).

**Table 1:** Participant demographics

Variable	%(*N*)
**Age group**	
16–25	12.5 (2)
26–40	56.3 (9)
41–60	18.8 (3)
61+	12.5 (2)
**Sexuality**	
Heterosexual	68.8 (11)
Gay	25.0 (4)
No answer given	6.3 (1)
**Relationship status**	
Single	25.0 (4)
Partnered	43.8 (7)
Married/de facto	25.0 (4)
Separated/divorced	6.3 (1)
**Employment**	
Full-time	50.0 (8)
Part time/casual	25.0 (4)
Unemployed/retired	18.7 (3)
Student	6.3 (1)
**Education**	
High school	25.0 (4)
Trade/certificate/diploma	18.8 (3)
Undergraduate degree	31.3 (5)
Postgraduate degree	25.0 (4)
**Helpline used**	
Lifeline (13 14 11)	50.0 (8)
Beyond Blue (1300 22 4636)	50.0 [8
Mensline (1300 78 99 78)	25.0 (4)
Headspace/eheadspace (1800 650 890)	25.0 (4)
Suicide Call Back Service (1300 659 467)	6.3 (1)
1800 Respect (1800 737 732)	6.3 (1)
Q Life (1800 551 800)	6.3 (1)
Mind Australia Carer Helpline (1300 554 660)	6.3 (1)
“Gambling helpline” *	6.3 (1)
“Drug and Alcohol line” *	6.3 (1)
**Time since last helpline use**	
In the last week	12.5 (2)
In the last month	6.3 (1)
In the last three months	12.5 (2)
In the last year (> 3 months)	37.5 (6)
More than a year ago	31.3 (5)
**Number of helplines utilised**	
1	43.8 (7)
2	31.3 (5)
3	18.8 (3)
4	6.3 (1)

*Note* *Response described verbatim

### Theme 1 – (In)authenticity: securing connection on the call

The importance of connection between men and their telephone counsellor was evident across participant responses, with the counsellor’s ability to quickly build rapport, establish trust and make men feel comfortable seen as essential to a meaningful and helpful caller experience. Men spoke both about counsellor attributes or approaches that caused fractures in their relationship with the counsellor and identified suggestions for strategies that may help counsellors to create and sustain connection with male callers.

#### Contributors to disconnection

Thirty-four-year-old Henry reflected that he viewed this connection as the cornerstone of the interaction:

*Your (the counsellor) main focus should be connecting with that person, making sure that person is feeling that he or she’s not alone in this, and that he or she is actually speaking to a human, not just a random on the other side of the line*

Establishing such connection was understood as potentially challenging for telephone counsellors, with men describing situations where they felt disengaged and estranged from the interaction. An emotionless tone of voice could be perceived as disinterest and serve as an immediate barrier to connection, carrying the potential to make men feel burdensome and unwelcome at the service. For example, 71-year-old Phil perceived a lack of warmth and empathy from his counsellor which generated a sense that he was just another somewhat typical caller:

*Tone of his voice. It just sounded like it was another… like I’d rung up to make a grocery order or something, he was just writing down… Okay. Pound of potatoes… Maybe I was expecting too much. I don’t know*

The service requirement for counsellors on some helplines to refrain from self-disclosure during a call may have contributed to the disengagement for some participants. For example, 34-year-old Henry felt that his counsellor not offering up their first name during the opening interaction impeded connection and communicated a sense that the help was somewhat anonymous and perhaps generic. Henry highlighted the discord between his need to fully connect with another person in his time of distress with the distance he experienced from the counsellor:

*Because I felt the need to connect with someone in that phone call, I vividly remember that I asked, “Oh, my name is so and so. What is your name?” And the response back was saying, “Oh, we’re not allowed to give up our names.” It just made me feel very disconnected because I needed to connect with a human being*

He went on to elaborate on the consequences, suggesting he was left feeling “*confused, frustrated, and I think very… I was already feeling anxious, but I think it didn’t help. There wasn’t any… at any point in time during that phone call, that the anxiety lifted, or I felt heard”.*

An overreliance on prescriptive phrases or responses led some participants to feel as though they were receiving generic rather than tailored help. For 39-year-old Will, this resulted in the perception that the interaction was somewhat contrived. This created feelings of detachment and difficulties in establishing the trust he needed to work with the counsellor:

*I wasn’t speaking to somebody who was actually going to give me support and guidance on what to do. I could make the joke and say, “Whatever you’re reading from, just email that to me and we’ll move on”*

Here, Will’s assertion that the counsellor’s script could have been emailed with the same effect (and perhaps affect) rendered the interaction somewhat formulaic without spontaneity or collaborative avenues between the caller and counsellor.

Connection could also be ruptured by counsellors’ response to identified safety or suicide risks. For example, 29-year-old Joe’s experience of being recommended to call an ambulance upon disclosure of suicidal thoughts led to feeling misunderstood amid forfeiting opportunities to explore the factors contributing to his suicidality on the call:

*That kind of triaging sort of feels like someone going into the emergency room without asking you like, “Oh, where’s the pain?” Or “What’s actually wrong?” And yeah, that’s been frustrating in the past. Those are times where I maybe end the conversation not feeling any better*

Failing to secure connection had the potential to increase distress, and to decrease men’s likelihood of utilising crisis helplines in future times of crisis. For 71-year-old Phil, who had a history of complex trauma and trust issues, his helpline experience served to reinforce internal beliefs about his lack of worthiness of others’ time and care:

*Look, I’m not going to contact anyone in future. I feel like it’s a waste of time and it just makes my emotional attachment get worse. No one cares. I don’t think anyone cares. They pretend they care. Well, I don’t say pretend, that’s the wrong word. They appear to care, but they don’t. That’s my honest opinion. And that’s what I’m stuck with. I wouldn’t bother ringing again*

#### Strategies to build connection

Participant responses outlined strategies and approaches that may help to overcome feelings of disconnection and allow counsellors to actively work to build rapport with callers. First, participants felt that there was scope for counsellors to bring more of themselves to the interaction, engaging in reciprocity to facilitate open conversations and humanize the exchange. For example, 46-year-old Andrew shared his recommendation for counsellors to *“just be yourself. And that’s all someone’s calling for, is to have an honest conversation with you and maybe get what’s off their chest. But just genuineness, like just be genuine, be yourself. Put your personality into the call and work.”*

Some participants highlighted the need for counsellors to recognise the individuality of male callers, and in some cases tailor their language, tone, or approach to suit each caller. Nineteen-year-old Lucas reflected that he found the service he called to be impersonal. He suggested that increased time spent getting to know and understand each caller would help counsellors provide tailored and person-centred support:

*Part of it is about identifying the person that you are on a call with when you can’t see them, and you only have a small amount of time to speak to them. If someone had an extra five minutes just to hang out with me, then they might be that much better equipped to give advice*

33-year-old Yuan, who had used a crisis helpline service multiple times, reflected that he felt the connection would have been strengthened through speaking with a gay male counsellor. He suggested a need for increased diversity within the helpline workforce to facilitate matching callers and counsellors to aid empathy:

*To begin with I didn’t (think about the counsellor’s gender), but three out of three, all just ladies, I thought that was… I would probably feel better if, not just talking to a guy, but perhaps if I could talk to a gay guy…if there are actually more people who we feel like we can identify with from the other side of the call, then it will make it a less difficult time, put it that way…I think they could probably employ more people that are men, and also from more diverse background*

Rapport building through acquiring information about a man’s personality, interests, and life outside of their specific challenges or concerns was also suggested as an important way to build connection, generate a sense of care, and alleviate distress through allowing the caller to focus on life contexts, as reflected by 29-year-old Joe:

*There have been times where someone’s just picked up on some minor detail of something I said. And then, maybe they’ve spun that out into a joke or a question about my work or my family, or a reference. Or someone’s asked me what I’m studying and they’re like, “Oh, what do you like about that?” Sort of that kind of, bring you off this tempo. Make you think about the broader dimensions of your life, I’ve found that to be effective. Just reminding you that there are lots of things outside of this cloud that you have*

Further, 34-year-old Henry felt that validating of the man’s experience through reflective listening and paying attention to the specificities of each caller’s story may reduce their perceptions of generic or scripted counsellor responses:

*Oh, I can see why you’re feeling sad because this happened. You shouldn’t be so hard on yourself because you did this… Actually reaffirm what you hear from that other person just to validate what they’re feeling, specific to their circumstances*

### Theme 2 – men’s expectations of the counsellor’s role in facilitating outcomes

Extending beyond securing connection, men spoke about their expectations of the helpline and how they viewed the role of the counsellor in facilitating call outcomes. Men’s responses detailed their journeys through crisis helpline calls, providing insight into what kind of support men were looking for from the interaction, and whether their needs were met. Important in this theme was the finding that most men felt supported when there was a balance between having an opportunity to tell their story, generating a feeling of being heard, and an action-oriented pathway forward, generating hope. The counsellor’s ability to recognise and attend to these fluctuating needs impacted the level of satisfaction felt by men.

#### Space to talk it out

Many men, including 27-year-old Li spoke of the call initially as a space to vent, identifying the counsellor as a ‘sounding board’ that allowed them to untangle and make meaning of their situation:

*Me explaining the situation I think in my own words also helped me articulate how I was feeling… I think when you’re going through that and when your mind is a bit of a mess, it helps when you articulate it to someone else yourself. I think that was the main positive for me*

Time spent voicing their issues, potentially for the first time, allowed men to clarify the depth of their challenges, acknowledge associated emotions, and begin to see potential steps forward.

While the kind of talk facilitated by crisis helplines serves a useful purpose, 65-year-old Stewart’s account spoke to the potential for discussion to open *‘a tin of worms’* resulting in increased distress. This highlights the role counsellors have in providing containment, orienting callers to the helpline environment, educating about the bounds of service provision (e.g., time limits), and ensuring that expectations are realistically mapped and agreed upon:

*A couple of minutes into the conversation, I thought I had probably engaged very well with the person, and this is going to get somewhere practical… And then it probably finished abruptly, only from a time constraint…then I was probably dealing with a lot of raw emotions post the contact…I would’ve been… yeah, a bit more miserable, I suppose. And that wasn’t good enough for me, because it actually, because the conversation that I would’ve had… it would’ve triggered a lot of issues. So then potentially, I was in a worse situation post engagement, than I was pre-engagement*

Stewart went on to share about his experience, *‘that’s affected me re-engaging again, which I haven’t done*.’ This account demonstrates the complexities of supporting men to self-disclose within the time-limited contexts of helpline calls, and the risk posed by that temporality and inability to follow up (or follow through) with specific counsellors.

Some men indicated the need for counsellors to play an active role in facilitating disclosure through the use of directive questioning and guidance to help the man identify the issue of focus for the call, rather than relying solely on open-ended questioning techniques. 29-year-old Joe discussed the differing levels of comfort men may feel making a call versus openly offering disclosures and provided suggestion of how counsellors might assist male callers who are reluctant or unable to identify and disclose key issues:

*I feel like they (men) would need to be given prompts, a bit of a shopping list. It’s like, “Hey, mate sounds like you’re having a rough time. Glad you called. Is it work? Is it this? Is it that? Is it money? Yeah. Are you in a relationship?” Just because I mean to pick up the phone is one thing, but to actually crack open the walnut, I think is pretty difficult. And sometimes, I don’t know, that’s really got to be coaxed out*

#### Moving from talk to action

While initial communication of the issue was essential, many men felt that this alone was not enough to constitute a helpful call, with 33-year-old Yuan sharing ‘*listening is great, but what’s after listening, I think there has to be something that’s more helpful than just listening’.*

Many men noted that they called a helpline to receive advice or solutions, hoping for a practical problem-solving approach to their issues. 46-year-old Andrew referenced such support was required as his distress stalled his ability to identify or navigate a path forward:

*At the time, in my situation, I needed practical advice. I needed someone who was thinking clearly to say, you need to do this. This is the route you need to go if you want to get help. Because yeah, I was in no state of mind to be making those decisions for myself*

Twenty-three-year-old Rishi was also seeking advice but felt that the options offered to him during his phone call were not particularly helpful as they didn’t reflect his own personal experience. Here, he shared that a personalised and step-by-step plan would have helped him to follow through with seeking further support at the end of the call:

*If they were genuinely interested in giving me very specific solutions, and step by step, I think that would be helpful for me. Because if they would’ve been action oriented, I would have definitely taken some action about it*

While many men reinforced the importance of helplines offering encouragement of further resources or support, 27-year-old Bryn recounted that at the time, he was not ready to accept and take on that advice:

*I think they did a good job of being there and listening and like I said, I mean, encouraging to seek out further support is good. Like I said, I didn’t really take that advice to heart at the time for the variety of reasons, but that’s as much on me as it is on them. Obviously, you can’t force someone to do something if they’re not ready to*

Although he said there was nothing the counsellor could have done to better encourage future help-seeking, Bryn did reflect later in the interview that his helpline use had eventually reduced the barriers to accessing psychological care, demonstrating the potential long-term bridging of the crisis call experience:

*It’s led me to see a psychologist regularly nowadays. It was the first barrier to get to that point I guess*

For some participants, their contact with a helpline enabled them to access support and started them on a recovery or support journey, reinforcing the value of helpline services for men as an accessible entry point to services. Participants noted the importance of counsellors being knowledgeable in a wide range of culturally relevant and tailored resources or referral services, for example 34-year-old Isaac shared satisfaction when the helpline counsellor considered his religious background when suggesting referral resources:

*I actually told them about my religious nature, and I was directed to meet a counsellor who was… in my local church, so it was a double win for me, because I actually got someone who was here to assist me in my spiritual upliftment, and also in my mental wellbeing*

Finally, some participants felt that it was important for the counsellor to recognize and check in with them about what they needed at that time, to orient the interaction towards that form of support. Twenty-even -year-old Li suggested, *I think one of the things that could help is if the worker were to ask the individual that was calling like, ‘do you need me to listen to you? Or do you need me to (help with) a solution.’* As discussed by Li, this may present a way for counsellors to ensure that the man and counsellor are aligned on the goal of the interaction and positions the man as an autonomous expert with control over the situation.

## Discussion

The present study explored Australian men’s experiences of crisis helplines, providing insight into factors influencing engagement. We found that men viewed the primary role of the counsellor as establishing a secure connection through displaying warmth and authenticity, building a foundation for the rest of the call. Following initial rapport building, men detailed the ways in which productive outcomes could be achieved through providing space for narrative storytelling and aiding in referrals to further support services when required. Findings contribute to the existing literature on men’s helpline use, reinforcing their value as a suicide prevention service while concurrently identifying target areas to improve men’s engagement and outcomes.

Men emphasized the importance of establishing an authentic connection with their counsellor as early as possible in the call, with failure to do so leading some men to disengage from the call without receiving the support they sought. This finding echoes the reflections of Australian therapists that failure to secure rapport with male clients in the first session could lead to those clients dropping out prematurely [33]. Here, the callers’ control over the interaction and ability to terminate the call removes opportunity for counsellors to attempt to repair ruptures in the relationship, reaffirming the critical importance of securing and maintaining an authentic connection with male callers.

How such an authentic connection could be reliably established is a considerable challenge for crisis helplines. While standardized frameworks and protocols exist for crisis helpline counsellors to ensure service fidelity, restrictive adherence to these protocols appears to result in some male callers feeling that they are simply being read a script of generic responses. Participants lamented the challenges of receiving a personalised experience through a generalized and anonymous service. For example, one participant’s wish that counsellors could provide names (even pseudonyms) indicates that some reciprocity may be required to secure an authentic connection with male callers. That is, participants in the present sample may have needed to sense that they are being heard by another *person*, rather than a disembodied voice. Indeed, Mishara and colleagues’ study regarding counsellor behaviours and associated outcomes found that counsellors’ self-disclosure of personal experiences (when done to convey understanding towards the help-seeker) was associated with positive outcomes [34]. While the practice of not sharing personal experience stems from prominent theories of psychotherapy, Mishara and colleagues argue that this may not translate to the telephone crisis service context, and that some personal disclosure by counsellors may aid in compensating for the anonymous nature of helpline calls [34]. Previous qualitative research with frequent callers to Lifeline has also reported caller’s recommendation that helpline counsellors disregard the ‘rules’ and disclose more information about themselves, including pseudonyms [35]. These findings present an opportunity for services to explore how counsellors may introduce some level of personal disclosure in their crisis interactions to convey to the help-seeker genuine and authentic interest and connection.

Supporting previous literature, participants used crisis helplines both as an avenue to discuss and seek emotional support for their concerns, and to gain knowledge about additional resources or coping strategies [18, 26]. Our results offer some level of depth into *how* these needs may be best met within the helpline context. Many participants spoke of utilising the counsellor as a ‘sounding board’ supporting Feo and LeCouter’s assertion that for some men, the purpose of calling helplines may be just to talk without seeking a specific solution [26]. However, several men expressed wanting a more solution-focused or practical approach than they received from the helpline, at odds with the premise of many helplines in providing emotional support which aims to empower callers to identify their own strengths, resources, and pathway forward using nondirective counselling techniques [36].

Clearly the provision of problem-focused coping strategies is an identified need for many men, and as such, services should consider ways to incorporate this kind of support within the context of helplines. Indeed, research regarding men’s engagement in psychotherapy indicates that in general, men prefer a transparent, collaborative approach with a goal or action-oriented structure (33). The focus on nondirective counselling techniques in helplines has been previously questioned, as some research indicates that collaborative problem-solving, including at times direct suggestions of action plans, facilitates increased positive outcomes among callers [37]. Attuning to the help-seekers needs and knowing when to apply directive versus non-directive strategies represents a key challenge, noted by one participant who suggested counsellors ask the caller to identify the kind of support they are after. One way to balance the requirements of helplines with the needs of solution-focused male callers may be through increasing helplines’ databases of male-sensitised support services for referrals. Specifically, including knowledge of informal supports such as men’s community groups and activity-based avenues rather than solely focusing on medical or mental health services might be beneficial, as research indicates many men prefer informal supports [38].

### Implications: towards gender-sensitive crisis helpline service delivery

These findings provide new depth and nuance into the experiences of men using crisis helplines and carry important implications for the helpline sector. First and foremost, findings highlight the importance of the relationship between caller and counsellor and suggest that more can be done to ensure helpline counsellors have the skills and confidence to connect and establish trust with male callers. Based on prior work within the context of psychotherapy, we see an opportunity to improve male caller outcomes by tailoring crisis helplines towards the needs of male callers. Previous research indicates that in general, medical and mental health training programs pay inadequate attention to masculinities and their interconnections with men’s engagement with services [12, 39]. Some empirical evidence suggests that mental health clinicians without understanding of masculine socialisation impacts render poorer outcomes with male clients [40], and that tailored training in engaging male clients has the potential to improve clinical competencies [41]. While the above research primarily focuses on therapists, helpline counsellors engaging with men would also likely garner significant benefits through these skills. The crisis helpline setting carries inherent challenges in sensitising intervention for specific population groups, given the time and information limited context, a predominantly volunteer workforce, and a generalised approach where counsellors are trained to apply non-judgemental approaches regardless of the caller’s demographics [42]. Consequently, we argue that training within the crisis helpline setting requires specific adaptations responsive to the challenges of this context, developed in partnership with helpline services. Finally, while many of the approaches noted by participants reflect core tenets of helpline care (and support more broadly) such as building rapport, using micro skills of reflection and validation, and providing tangible pathways forward, building gender awareness of helpline counsellors has the potential to increase understanding of *how* these skills may be best applied with male callers. Our research findings have identified target areas for improvement in engagement with male callers that can inform the development of such a training, putting the perspectives on male callers at the forefront of intervention development. Our findings also demonstrate a need for helpline services to invest in educating men in the community about the scope of helpline services including strengths and limitations of services, in order to manage expectations and demystify the process of calling a helpline.

### Limitations and future directions

While this study provided insight into an under-researched area, it had some limitations. Firstly, participants had utilized a variety of helpline services, and responses here relate to their general experiences and are not service specific. This broad inclusion criteria allowed us to learn about men’s experiences with a wide range of helplines but meant that the implications for improving individual services are unclear. Second, it is likely that recruitment strategies utilised (e.g., social media advertisements) and the nature of the interview methodology implemented resulted in a sample of men with greater comfort and motivation in sharing their experience, thus missing the views of men who are reluctant to engage with health research. Future attempts to recruit male helpline users diversely (i.e., invitations following helpline calls) and triangulate data with provider perspectives might extend the current findings. Thirdly, it is possible that potential participants chose not to express interest due to the use of Zoom. While participants were not required to use the video feature of Zoom, future research might benefit from making this flexibility explicit in recruitment materials. Finally, since the interviews concerned men’s experiences of crisis helplines in Australia, the findings may not reflect the experiences of men accessing helplines in different cultural and geographical settings. We recommend that similar research be conducted in these settings to better understand men’s crisis support needs.

## Conclusion

This study represents the first to qualitatively explore men’s experiences of crisis helpline services, providing much-needed information on how men view and interact with helpline counsellors. Men emphasised that building an authentic connection with their helpline worker was crucial to attending to their needs and facilitating desired outcomes. Building on research in men’s engagement in therapy, we argue that upskilling helpline counsellors to engage effectively with men will have beneficial impacts on male caller outcomes.

### Electronic supplementary material

Below is the link to the electronic supplementary material.

Supplementary Material 1

Supplementary Material 2

Supplementary Material 3

Supplementary Material 4

## Data Availability

The data generated and analysed during the current study are not publicly available due to ethical restrictions concerning personal and sensitive information shared by participants. Please direct enquiries regarding data used in this study to the corresponding author.
