# Supplementary material for: “Put your personality into the call”: A qualitative interview study illuminating strategies for improving men’s engagement on crisis helplines
Source: BMC Public Health. 2024 Jun 27;24:1720. doi: 10.1186/s12889-024-19242-x (PMC11212170; doi:10.1186/s12889-024-19242-x)
Supplement: Supplementary file 2 — Supplementary Material 2 [file 12889_2024_19242_MOESM2_ESM.docx]

**Template analysis- 24 May 2022**

1. (In)authenticity: a barrier to genuine connection
   1. Starting on the wrong foot – brief window for connection
   2. Counsellor demeanor- tone of voice
   3. Lack of personalisation - not identifying with the man as a unique caller
      1. Providing scripted and generic responses
      2. Validation of the unique situation
      3. Validation of the man- affirm identity and meaning
2. Counsellor role of facilitating outcomes
   1. A sounding board- space for men to vent and process
   2. Hope generated through action
      1. In the immediate- reframing solutions to emotional management/coping strategies
      2. In the medium/long term -identifying and encouraging specific and relevant further supports
   3. Striking a balance
      1. Feeling heard is not enough for satisfaction
      2. Rushing to intervene results in lack of validation

**Template analysis 2 – 4 October 2022**

1. (In)authenticity: a barrier to genuine connection
   1. A fragile connection
   2. Helper communication style
   3. Personalising the experience
2. Counsellor role of facilitating outcomes
   1. A sounding board
   2. Hope generated by action
   3. Striking a balance

**Final template – 5 December 2022**

1. (In)authenticity: securing connection on the call
   1. Contributors to disconnection
   2. Strategies to increase connection
2. Men’s expectations and the counsellor’s role in facilitating outcomes
   1. Space to talk to out
   2. Moving from talk to action
