# Supplementary material for: “Put your personality into the call”: A qualitative interview study illuminating strategies for improving men’s engagement on crisis helplines
Source: BMC Public Health. 2024 Jun 27;24:1720. doi: 10.1186/s12889-024-19242-x (PMC11212170; doi:10.1186/s12889-024-19242-x)
Supplement: Supplementary file 3 — Supplementary Material 3 [file 12889_2024_19242_MOESM3_ESM.docx]

**Men’s experiences of crisis helplines: Interview schedule**

1. Think back to a time that you called a helpline. What was happening for you in your life at that point?
2. Can you talk me through the experience of calling a helpline?
3. How did you feel when speaking with the helpline worker?
4. To what extent did you feel listened to?
   1. What did the worker do or say that told you that you were/were not being listened to?
5. To what extent did you feel welcome at the service?
   1. What did the worker do/say that made you feel welcome/unwelcome?
6. What was your reason behind calling a helpline? For example, was it to get help with solutions to your problems, to access other services, or just to talk to someone?
   1. What were you needing from a helpline worker at that time?
   2. Did you feel satisfied that the call had met your needs in that point in time?
   3. If not, what could the helpline worker have done differently/additionally to help meet your needs?
7. After your most recent/prominent call, how did you feel? What (if any) actions did you take?
8. What worked well for you when calling a helpline?
9. What could have been improved about your experience using a helpline?
10. What was your attitude towards helplines before you first used them?
    1. Has this attitude shifted since using them?
11. Do you think you would use helplines again in the future if you found yourself in a similar situation?
12. Would you recommend a helpline to a mate?
    1. Why/why not?
    2. When/under what circumstances would you recommend a helpline to a mate?
13. How do you feel that mental health helplines could improve their service to meet the needs of callers like yourself?
14. Anything else to add?
