# Supplementary material for: “Put your personality into the call”: A qualitative interview study illuminating strategies for improving men’s engagement on crisis helplines
Source: BMC Public Health. 2024 Jun 27;24:1720. doi: 10.1186/s12889-024-19242-x (PMC11212170; doi:10.1186/s12889-024-19242-x)
Supplement: Supplementary file 4 — Supplementary Material 4 [file 12889_2024_19242_MOESM4_ESM.docx]

**22987 - Clinical safety protocol**

A comprehensive and detailed suicide risk assessment and management protocol has been developed. This protocol is described below and is also outlined in Figure 1. It details the assessment and management of clinical risk identified in research interviews.

In the event that current suicide ideation is disclosed during interview, or the research assistant becomes concerned about a participant’s wellbeing, the participant’s level of suicide risk will be assessed face-to-face (by the study research assistant; RA) during the research interview over Zoom. Further, the research assistant will perform a ‘check in’ with all participants at the conclusion of the interview to assess for any risk and refer to support services as required. A senior clinical supervisor will be available on call should the RA require ad hoc consultation and supervision.

In the case of distress identified other than suicide risk, participants will be provided with the resources available on the PLS and encouraged to seek support. The research assistant conducting interviews will pause the interview if any distress is observed, and remind participants that they are able to pause or stop the interview at any time. Participants will be offered opportunity to debrief with their in-shift supervisors (in the case of TCSs) or a registered mental health professional on the research team if required. Aside from emotional distress, no other risks are expected due to participation in the research.

***Definitions of “suicide risk”***

Participants’ level of suicide risk will be classified as one of three categories. These categories, and their corresponding responses are outlined in Figure 1 below, and are based on the expert consensus guidelines developed for Project Synergy (Robinson et al., 2016). The suicide risk categories that feature in the guidelines were originally described by Joiner, known as an international expert in suicide prevention (Joiner et al., 2007)

- **High risk:** Suicidal ideation, suicidal capability (i.e. a sense of fearlessness or competence to make an attempt, availability of means and opportunity, specific plan, or preparing) and suicidal intent are all present, and the risk is imminent. For example, a participant who indicates they are in the process of making a suicide attempt or expresses suicidal ideation and cannot assure their safety would always be considered “high risk”.
- **Moderate risk:** Suicidal ideation and *either* suicidal intent *or* suicidal capability are present. If protective factors are absent, this situation may be considered high risk. For example, a participant who expresses suicidal ideation and has a plan but their intent is low, or expresses suicidal ideation and intent but does not have a plan or access to means, should be considered “moderate risk” (unless protective factors are absent).
- **Low-moderate risk:** *Either* suicidal desire, suicidal intent *or* suicidal capability are present. If there is an absence of protective factors, this situation may be considered moderate risk. For example, a user who expresses suicidal ideation but has no plan or intent to act should be considered “low risk”, unless they express low perceived social support or lack of purpose in life, and/or are not engaging with the relevant professional.

**Documentation and communication between researchers**

All risk assessments carried out and actions taken in response will be thoroughly documented as required by standard operating procedures at University of Melbourne. Any adverse events will be submitted via incident reporting through the Infonetica ERM system.

Clinical response by research assistant or supervisor

***User determined to be at HIGH RISK of suicide***

- Ask participant to provide their current location address
- Contact crisis/emergency services (ambulance, police)
- Take all reasonable steps to ensure the participant’s safety
- Inform the participant of the steps that will be, or have been taken
- If possible, maintain contact with the user until assistance arrives

***User determined to be at MEDIUM RISK of suicide***

- Encourage the participant to contact support services for assistance
- Encourage the participant to speak to a person in their location who could provide support
- Work collaboratively with the user to help keep them safe or seek emergency assistance

***User determined to be at LOW RISK of suicide***

- Encourage the participant to contact support services for assistance
- Ask the participant whether there is a person in their location who could provide support

RA detects possible clinical risk via research interview

RA conducts face-to-face risk assessment via Zoom

Inform project supervisor (SR)

Risk assessments and actions carried out appropriately documented and reported back to the project supervisor

Figure 1. Safety protocol
